# The Emerging Role of Long Non-Coding RNAs in Development and Function of Gilthead Sea Bream (*Sparus aurata*) Fast Skeletal Muscle

**DOI:** 10.3390/cells11030428

**Published:** 2022-01-26

**Authors:** Isabel García-Pérez, Anna Molsosa-Solanas, Miquel Perelló-Amorós, Elena Sarropoulou, Josefina Blasco, Joaquim Gutiérrez, Daniel Garcia de la serrana

**Affiliations:** 1 Department of Cell Biology, Physiology and Immunology, Faculty of Biology, University of Barcelona, 08028 Barcelona, Spain; isabelgarcia@ub.edu (I.G.-P.); anna.molsosa@gmail.com (A.M.-S.); miquelperelloamoros@gmail.com (M.P.-A.); jblasco@ub.edu (J.B.); jgutierrez@ub.edu (J.G.); 2 Institute of Marine Biology, Biotechnology and Aquaculture, Hellenic Centre for Marine Research, 71003 Crete, Greece; sarris@hcmr.gr

**Keywords:** lncRNA, muscle development, gilthead sea bream

## Abstract

Long non-coding RNAs (lncRNAs) are an emerging group of ncRNAs that can modulate gene expression at the transcriptional or translational levels. In the present work, previously published transcriptomic data were used to identify lncRNAs expressed in gilthead sea bream skeletal muscle, and their transcription levels were studied under different physiological conditions. Two hundred and ninety lncRNAs were identified and, based on transcriptomic differences between juveniles and adults, a total of seven lncRNAs showed potential to be important for muscle development. Our data suggest that the downregulation of most of the studied lncRNAs might be linked to increased myoblast proliferation, while their upregulation might be necessary for differentiation. However, with these data, as it is not possible to propose a formal mechanism to explain their effect, bioinformatic analysis suggests two possible mechanisms. First, the lncRNAs may act as sponges of myoblast proliferation inducers microRNAs (miRNAs) such as *miR-206*, *miR-208*, and *miR-133* (binding energy MEF < −25.0 kcal). Secondly, *lncRNA20194* had a strong predicted interaction towards the *myod1* mRNA (ndG = −0.17) that, based on the positive correlation between the two genes, might promote its function. Our study represents the first characterization of lncRNAs in gilthead sea bream fast skeletal muscle and provides evidence regarding their involvement in muscle development.

## 1. Introduction

Gilthead sea bream (*Sparus aurata*) is one of the most cultivated species in the Mediterranean area, whose production has already exceeded wild-fish captures [1]. Although this species has been extensively studied due to its commercial interest, more research is still needed to understand some physiological aspects better, such as the molecular networks involved in muscle growth control and development. As with most teleosts, gilthead sea bream shows indeterminate growth with continuous muscle accretion throughout its life thanks to processes of hyperplasia (i.e., recruitment of new muscle fibers) and hypertrophy (i.e., increase in fiber size) [2,3]. These two processes are the result of increased myogenesis, a complex process involving the coordination of several molecular networks, including the myogenic regulatory factors (MRFs), a group of transcription factors that play a key role in the control of the myogenesis [3,4]. In the onset of myogenesis, the myogenic factor 5 (Myf5) and the myogenic determination factors (Myods) are required for the determination of the myogenic lineage and the proliferation of the muscle satellite cells. Then, the myogenin (Myog) and the myocyte enhancer factor 2 (Mef2) lead to myoblasts fusion and differentiation, and the myogenic factor 6 (Myf6/Mrf4) is responsible for myotubes maturation. In the myoblasts fusion process, different membrane proteins are also involved [5,6], including the cadherins (Cdhs), which mediate cell–cell adhesions [7]; the caveolins (Cavs), implicated in vesicular trafficking and signal transduction [8]; and a recently discovered protein duo, Myomaker (Mymk) and Myomixer (Mymx), involved in plasma membrane hemifusion or fusion pore formation, respectively [9,10]. Besides, the Crk adaptor proteins (Crk and Crkl) and the dedicator of cytokinesis (Docks) are crucial members of the intracellular signaling networks that trigger myotubes formation [11].

Although it has traditionally been supposed that most genetic information is transacted by proteins and that RNA only plays an intermediary role, in the last decades, it was demonstrated that a majority of genomes from complex organisms are in fact transcribed into non-coding RNAs (ncRNAs) [12]. The term ncRNA refers to RNAs that are not translated into a protein but have a wide spectrum of functions in many cellular processes, both in physiological and pathological situations and are crucial in controlling transcription of other genes [12,13,14].

The long ncRNAs (lncRNAs) are a class of ncRNAs characterized by having a length ranging from 200 nt to 100 kb [15]. They can exhibit a wide or tissue-specific expression, and their transcription is usually lower than those of protein-coding genes [15]. Although the lncRNAs do not normally code for proteins, a fraction of putative small open reading frames (sORFs) were found in some lncRNAs that are translated into micropeptides [16,17], as in the case of Mymx [10,17,18]. Besides, lncRNAs play a fundamental role in the fine control of gene expression, acting at different levels: modulating chromatin structure, acting upon transcription factors binding, controlling RNA splicing, regulating translation, modifying mRNA stability, or interacting directly with proteins [13,19,20]. LncRNAs can also interact with microRNAs (miRNAs), another type of ncRNAs involved in posttranscriptional regulation of protein-coding genes by mRNA cleavage, translational repression, or mRNA destabilization [21]. LncRNAs can act as miRNAs precursors or as miRNAs sponges, thus altering their regulatory effect on mRNAs and introducing an additional layer of complexity in the miRNA-target interaction network [21,22,23]. All the specific interactions of lncRNAs are sometimes based on sequence complementarity, but it is suggested that, in many cases, the lncRNAs’ function is defined by their three-dimensional structure [24]. This might be the reason why the evolutionary conservation of the nucleotide sequence of lncRNAs is very low, and it is proposed that the secondary structure is conserved [24]. In some cases, where lncRNAs codify for micropeptides, such as the *mymx* gene, their sequence is conserved between species, but that only happens on very rare occasions [10,18].

Currently, most of the studies in the field of lncRNAs are limited to humans and model species showing that lncRNAs have key roles in glucose and lipid metabolism [25], the immune response [26,27], the occurrence and development of cancers [28], and the neural development [29], among many others. Moreover, several lncRNAs were described as important molecules that can also regulate myogenesis in mammals, although the function of the vast majority is not yet well defined [30,31]. They may be implicated in the maintenance of satellite cells pool, their activation, proliferation, differentiation, and self-renewal [32,33]. For example, *Linc-RAM* is a lncRNA that is specifically expressed in skeletal muscle tissue and promotes myogenic differentiation by interacting with Myod [34]. The lncRNA *Irm* regulates the expression of myogenic genes by binding to Mef2d, which in turn promotes the assembly of Myod/Mef2d on the regulatory elements of target genes [30]. The lncRNA *Munc* is also required for optimal myogenic differentiation since it induces the expression of *myod*, *myog*, and *myosin heavy chain 3* [35,36]. The fact that lncRNAs have such important roles in muscle development involves them not only in physiological conditions but also in pathological ones, such as dystrophy, atrophy, aberrant hypertrophy, or even in the recovery after an injury, in the process of necrosis, and muscle regeneration [16,32,33].

While the study of lncRNAs is progressing fast in humans and model species, information on teleost fish is scarce, and most studies are based on high-through sequencing platforms [37,38,39], providing valuable information on the molecular interactions between lncRNAs, mRNAs, and miRNAs. However, RNA-Seq studies can give us a limited picture of the lncRNAs functions due to RNA-Seq inherent limitations, such as the relatively small number of animals used and physiological conditions able to test. In addition, these studies of lncRNAs are of very little utility unless they are conducted in the same species of interest since the low conservation of lncRNAs sequences, even between closely related species, makes it difficult to translate findings on one species to another [24]. There are some recent studies in fish where the lncRNAs were reported to participate in many biological processes, including immune response [39,40,41], sex differentiation [42,43], smoltification process [44], intestinal homeostasis [45], and lipid metabolism [46]. Regarding the role of lncRNAs in fish muscle development and growth, there are very few studies that address this issue [37,47], and there is still much to explore in this field.

In gilthead sea bream, the mechanisms orchestrating the myogenesis and the molecular basis of muscle plasticity have only been studied from the perspective of protein-coding genes [4,48,49,50,51,52,53,54]. Hence, building on this research, the present work aims to identify lncRNAs with potential functions on the development and growth of fast skeletal muscle of gilthead sea bream. Therefore, existing transcriptomic data from the white muscle of this species were used to find expressed lncRNAs, and then the transcriptional profile of a subgroup was examined under different experimental conditions in which white muscle development and remodeling are expected.

## 2. Materials and Methods

**Figure 1:**
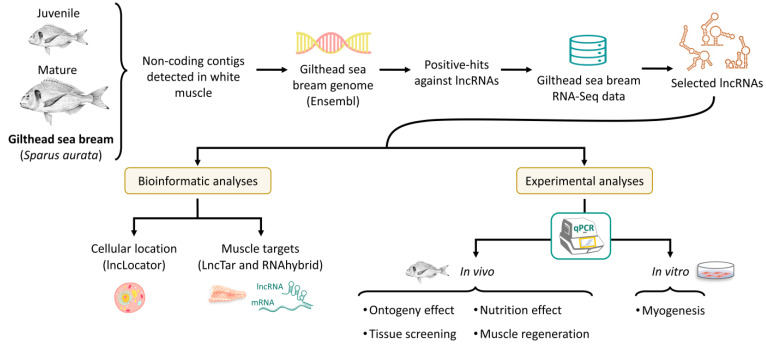
Workflow for the detection and analysis of gilthead sea bream fast skeletal muscle lncRNAs. Gilthead sea bream figure was extracted from https://www.fao.org/fishery/culturedspecies/Sparus_aurata/es (accessed on 15 November 2021).

Figure 1 shows the main steps followed for the identification and characterization of gilthead sea bream fast skeletal muscle lncRNAs. Specific databases and software used for each step are indicated in brackets.

### 2.1. Identification of lncRNAs in Gilthead Sea Bream

In order to detect lncRNAs expressed in the gilthead sea bream fast skeletal muscle, existent transcriptomic data obtained from publicly available GS FLX 454 normalized libraries were used [55]. The GS FLX 454 transcriptomes were blasted (BLASTn) against all lncRNAs annotated in the gilthead sea bream genome (http://www.ensembl.org/ (accessed on 20 November 2021)) using the BLAST2GO software (part of OmicsBox package v.1.0) [56]. The threshold for a contig to be considered a positive hit for a lncRNA was set at an e-value lower than 1 × 10^−90^ and similarity over 98%. The expression of identified lncRNAs was further investigated using fast skeletal muscle RNA-Seq data from mature and immature male gilthead sea breams (Figure 1).

### 2.2. RNA-Seq

The transcriptomic analysis was performed at the Institute of Marine Biology, Biotechnology, and Aquaculture of the Hellenic Centre of Marine Sciences (HCMR, Crete, Greece). The use of animals used for the transcriptomic analysis was approved by the relevant Greek authorities (National Veterinary Services) under the license No 32356 (AΔA: 984I7ΛK-K65). All procedures involving animals were conducted following the “Guidelines for the treatment of animals in behavioral research and teaching” [57], the Ethical justification for the use and treatment of fishes in research: an update [58], and the “Directive 2010/63/EU of the European Parliament and the council of 22 September 2010 on the protection of animals used for scientific purposes” (EU, 2010).

Briefly, the white muscle was sampled from non-mature (juveniles) and mature male gilthead sea breams. The total RNA was extracted with NuceloZOL (Macherey-Nagel, Duren, Germany) following the manufacturer’s instructions. RNA quantity was assessed by Nano-Drop ND-1000 spectrophotometer (NanoDrop Technologies, Wilmington, DE, USA). The quality of the extracted RNA was evaluated by agarose (1%) gel electrophoresis and by RNA Pico Bioanalysis chip (Agilent 2100 Bioanalyzer, Agilent, CA, USA).

Library construction and paired-end (PE) sequencing was carried out by Novogen (Novogene, UK). Muscle reference transcriptome and count matrix was generated applying Trinity software Trinity v.2.13.2 [59]. Differential expression was assessed using Bioconductor packages, including DESeq2 [60].

The RNA-Seq data were deposited in the European Nucleotide Archive (ENA) under the accession number PRJEB50017.

### 2.3. Prediction of Target mRNAs, miRNAs, and Cell Location of lncRNAs

LncRNAs were compared against the full cDNA sequences of 149 protein-coding genes and miRNAs known to be involved in muscle regulation (http://www.ensembl.org/ (accessed on 20 November 2021)) (Supplementary File S1). The mRNA target predictions were made using LncTar software v.1.0 (https://www.cuilab.cn/lnctar (accessed on 20 November 2021)) [61] with a normalized interaction threshold of ndG < −0.08. The potential interactions with mature miRNAs were investigated using RNAhybrid v.2.2.1 (https://bibiserv2.cebitec.uni-bielefeld.de/rnahybrid (accessed on 20 November 2021)) [62] with a minimal free energy (MFE) threshold of <−20.0 kcal. The subcellular location of lncRNAs was predicted using the lncLocator software v.1.0 (http://www.csbio.sjtu.edu.cn/bioinf/lncLocator/ (accessed on 20 November 2021)) [63] (Figure 1). MiRNAs sequences were obtained from the Ensembl database, and their mature sequences were predicted by alignment with known mature forms from zebrafish (*Danio rerio*) and tilapia (*Oreochromis niloticus*), downloaded from the miRbase database (https://www.mirbase.org (accessed on 20 November 2021)).

### 2.4. Experimental Trials

All animal-handling procedures were conducted following the Directive 2010/63/EU of the European Parliament and the council of 22 September 2010 on the protection of animals used for scientific purposes, the guidelines of the Spanish and Catalan governments, and with the approval of the Ethics and Animal Care Committee of the University of Barcelona.

Gilthead sea breams used in all the experiments were obtained from a commercial hatchery (Piscimar, Burriana, Castellón, Spain) and were acclimatized to the facilities at the University of Barcelona (Barcelona, Spain) for a minimum of two weeks prior to sampling or experimental manipulations. Fish were fed ad libitum twice a day with commercial pellets (Skretting, Burgos, Spain) and held at 23 ± 1 °C, a salinity of 35–37‰ and a photoperiod of 12 h light/12 h dark in a semi-closed recirculation system with a weekly renewal of 20–30%.

In order to infer the functions of the lncRNAs in the muscle, their transcriptional profile was analyzed in different in vivo and in vitro experimental conditions.

#### 2.4.1. In Vivo Experiments

For the exploration of the ontogeny effect on the expression of the lncRNAs, the epaxial white muscle tissue was collected from groups of 8 fish each of fingerlings (6.0 ± 0.5 g), juveniles (122.4 ± 2.3 g), and matures/adults (387.1 ± 41.9 g). For lncRNAs screening, the following tissues were extracted from 4 fish of 214.0 ± 12.1 g: white muscle, red muscle, skin, gills, eye, heart, adipose tissue, bone, brain, pituitary, spleen, stomach, proximal and distal intestine, liver, head kidney, pyloric caeca, and gonads. In order to analyze the effects of fasting and refeeding on lncRNA transcription, fish with an initial body weight of 50.0 ± 3.0 g were fasted for 21 days and then refed for 7 days. Samples of white muscle tissue were taken from 6 fish at the beginning of fasting and at the end of it at 0, 2, 5, and 24 h and 7 days after refeeding (as previously described in [64]). A muscle regeneration experiment was conducted to study the possible role of the lncRNAs in myogenesis after a muscle injury. Fish of 15.4 ± 3.5 g were used, and an injury was performed with a 2.108 mm diameter needle inserted vertically into the left epaxial muscle below the sixth radius to a depth of 1 cm. Samplings were performed at days 0, 1, 2, 4, 8, 16, and 30 after the injury. Each day, from 10 injured fish, a section of the muscle was removed from the left side (injured) and the right side, as self-control for each fish (for more details of this experiment, see Perelló-Amorós et al. [18]).

#### 2.4.2. In Vitro Myogenesis: Primary Myocyte Cell Culture

In order to infer the function of the lncRNAs during the process of myogenesis, their expression was analyzed in the different phases of a myocyte cell culture. This culture consists of a short period where they remain quiescent satellite cells (day 1), then a proliferative stage of the myoblasts (until day 4 of the culture), followed by a fusion phase of the myoblast to form early myotubes and their final maturation (from day 4 onwards) [4,65]. Six different cell cultures were performed following the protocol previously described by Montserrat et al. (2007) [65]. Cells were seeded at a density of 2 × 10^6^ cells per well in 6-well plastic plates (9.6 cm^2^/well) (Nunc, Labclinics, Barcelona, Spain). Cells were maintained at 23 °C and 2.5% CO_2_ in Dulbecco’s Modified Eagle Medium supplemented with 10% fetal bovine serum and 1% antibiotic–antimycotic solution. All media and reagents were obtained from Sigma-Aldrich (Tres Cantos, Madrid, Spain). The medium was renewed every 2 days during the culture. Samples were taken on days 2, 4, 6, 8, 10, and 12 after satellite cells seeding.

### 2.5. Primer Design

Primers for qPCR were designed from Ensembl lncRNAs sequences using Primer3 software v.0.4.0 [66] with a melting temperature of 60 °C. Primers, possible hairpins, or non-desirable primer-dimers were investigated using NetPrimer (http://www.premierbiosoft.com/netprimer/netprlaunch/netprlaunch.html (accessed on 13 November 2021)). Primers used in the present study are summarized in Supplementary File S2.

### 2.6. Gene Expression

#### 2.6.1. RNA Extraction and cDNA Synthesis

For RNA extraction from tissue, 40 to 500 mg of tissue (depending on tissue yield) were used, and total RNA was extracted with 1 mL of TRI Reagent^®^ Solution (Applied Biosystems, Alcobendas, Madrid, Spain). For RNA extraction from cells, the cells seeded in 3 replicate wells were pooled together at each sampling point during the culture, and total RNA was extracted with 1 mL of TRI Reagent^®^ Solution. The RNA concentration and purity of the samples were determined using the Nanodrop 2200TM (Thermo Scientific, Alcobendas, Madrid, Spain). The RNA integrity was checked in a 1% (w/v) agarose gel stained with SYBR-Safe^®^ DNA Gel Stain (Life Technologies, Alcobendas, Madrid, Spain). For cDNA synthesis, 1.1 µg of total RNA was treated with DNase I Amplification Grade (Life Technologies, Alcobendas, Barcelona, Spain) and retrotranscribed with the Transcriptor First Strand cDNA Synthesis Kit^®^ (Roche, Sant Cugat del Vallès, Spain) [49].

#### 2.6.2. Quantitative Real-Time PCR (qPCR)

The qPCRs were performed following the MIQE guidelines [67] in a CFX384TM Real-Time System (Bio-Rad, El Prat de Llobregat, Barcelona, Spain) using iTAQ Universal SYBR^®^ Green Supermix (Bio-Rad, El Prat de Llobregat, Barcelona, Spain). The analyses were carried out in triplicate, using for each reaction: 2.5 µL of iTAQ Universal SYBR^®^ Green Supermix, 1 µL of cDNA, 250 nM (final concentration) of forward and reverse primers, and 1.25 µL of DEPC water. The qPCR program consisted of 3 min at 95 °C, 39 × (10 s at 95 °C, 30 s at the annealing temperature of the primers and fluorescence detection), followed by an amplicon dissociation analysis from 55 to 95 °C with an increase of 0.5 °C each 30 s [49].

All the primers were first validated using a dilution curve with a pooled sample made before the analyses to confirm reaction specificity, efficiency of the primer pairs, absence of primer-dimers, and to determine the appropriate cDNA dilution to work with.

The mRNA transcript level of each studied gene was calculated relative to the geometric mean of the combination of the two most stable reference genes (*ef1a*: elongation factor 1 alpha, *rps18*: ribosomal protein s18, and *rpl27a*: ribosomal protein l27a) (confirmed by the geNorm algorithm) using the Bio-Rad CFX Manager™ software v.3.1, and following the Pfaffl method [68].

### 2.7. Statistics

All statical analyses and graphs were conducted using R-Studio v.1.1.419 [69] and ggplot2 [70]. Data normality and homogeneity of variance were estimated using Shapiro–Wilk and Levene’s tests. A Box-Cox transformation approach was used to transform non-normally distributed data and tested again on normality and homogeneity of variance assumptions. Differences between measurements were analyzed using a *t*-test or an ANOVA model followed by a Tukey’s post hoc test when homogeneity of variance was achieved. Pearson’s test was used to estimate the correlation between the transcription of the different genes studied.

Unless otherwise indicated, values are shown as mean ± SD. The signification threshold was established as the *p*-value (*p*) < 0.05.

## 3. Results

### 3.1. lncRNAs Selection

**Figure 2:**
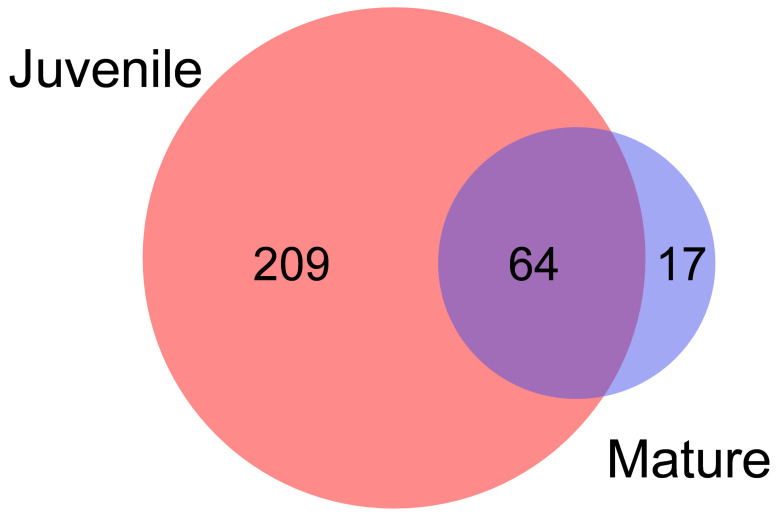
LncRNAs detected in fast skeletal muscle GS FLX 454 transcriptomes. Venn diagram representing LncRNAs identified in the GS FLX 454 transcriptomes of juvenile and mature gilthead sea breams from Garcia de la serrana et al. (2012) [55]. The number of lncRNAs found only in juveniles or matures is shown inside the individual circles, while lncRNAs shared are shown in the intersection.

Because many lncRNA can show low levels of the transcriptome, normalized GS FLX 454 transcriptomic data from juvenile and adult gilthead sea bream were used to detect as many as possible in the gilthead sea bream fast skeletal muscle [55]. A total of 290 lncRNAs were identified, with 209 found only in juveniles, 64 shared between juveniles and adults, and 17 expressed only in adults (Figure 2; Supplementary File S3).

LncRNAs of interest were ranked using their relative expression in the GS FLX 454 transcriptomes (data not shown). However, as it is a normalized transcriptome expression, data are not accurate; therefore, non-normalized RNA-Seq data from fast skeletal muscle of juveniles and adults of gilthead sea bream was used to estimate their abundance initially. The number of counts mapped for each of the lncRNAs was extracted and selected the 20 first lncRNAs in which at least one count was present in all the samples (Supplementary File S4). From those 20 selected, at least eight of them were already showing significant differences in the expression between juveniles and matures (Supplementary File S4). Primers were designed to amplify the selected 20 lncRNAs, with only 12 successfully amplified by qPCR. These 12 lncRNAs were named throughout the text as “lncRNA” plus the last five numbers of their Ensembl Transcript ID.

**Figure 3:**
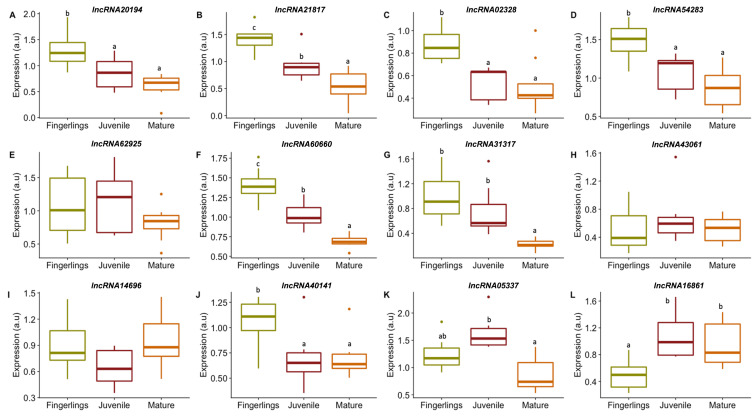
Expression of selected lncRNAs in gilthead sea bream fast skeletal muscle at different ontogenetic stages. Boxplots showing lncRNAs expression in gilthead fast skeletal muscle obtained from fingerlings (green box), juveniles (dark red box) or adults (orange box) (**A**–**L**). Gene expression is indicated as arbitrary units (a.u). Significant differences between ontogenetic stages are indicated as different letters when *p* < 0.05. Outliers are presented as points.

Our interest was focused on those lncRNAs that might have an active role in muscle development; therefore, the expression of the 12 lncRNAs candidates was studied in the fast skeletal muscle of gilthead sea bream fingerlings, juveniles, and matures (Figure 3). The *lncRNA20194*, *lncRNA21817, lncRNA02328, lncRNA54283, lncRNA60660*, *lncRNA31317,* and *lncRNA40141* were significantly more expressed in the fingerlings compared to matures and/or juveniles (Figure 3A–D,F–G,J). The *lncRNA16861* showed significantly lower expression in fingerlings compared to juveniles and matures (Figure 3L). The *lncRNA62925, lncRNA43061*, *lncRNA14696*, and *lncRNA05337* had similar transcription in the three ontogenetic stages (Figure 3E,H–I,K).

Since it was intended to study lncRNAs with relevant roles in muscle growth and development, those lncRNAs more expressed in the stages in which the fish growth rates are higher (fingerling and juvenile stages) had to be selected. Hence, *lncRNA20194*, *lncRNA21817*, *lncRNA54283*, *lncRNA60660*, and *lncRNA40141* were prioritized and selected to study their transcriptional profile under different physiological conditions further. Moreover, the *lncRNA16861* that had lower transcription in fingerlings compared to juveniles and matures, and the *lncRNA14696* that showed no differences between stages were selected to explore the transcription of lncRNAs with different patterns of expression. In addition, for the selection of lncRNAs, the expression level of each lncRNA was taken into account, and those with the highest expression were selected.

### 3.2. lncRNAs Subcellular Location and Targets

In order to elucidate the possible functions of the selected lncRNAs, their sequences were analyzed to determine their predicted subcellular location and their possible interactions with protein-coding genes mRNA and miRNAs related to muscle growth and development in other vertebrates (Table 1).

The *lncRNA16861, lncRNA20194,* and *lncRNA21817* were predicted to be in the cytoplasm, while *lncRNA14696* was predicted to be in the nucleus, all of them with a probability over 0.70 (Table 1). Three of the selected lncRNAs, *lncRNA40141*, *lncRNA54283*, and *lncRNA60660*, appeared to have a relatively low probability of being in the cytoplasm and the nucleus (Table 1).

Regarding the possible interactions between selected lncRNAs and genes known to be involved in muscle growth and development, it was observed that several lncRNAs showed some interaction with these genes (Table 1). However, only *lncRNA20194* appeared to have strong interaction with *myod1* (ndG < −0.170) (Table 1).

The analysis of possible interactions between selected lncRNAs and miRNAs revealed that all the lncRNAs selected in the present work interact with several miRNAs with an MFE lower than −20 kcal (Table 1). Some of the lncRNAs showed relatively strong interactions (MFE < −25 kcal) with different miRNAs, as in the case of *lncRNA14696* (*miR-133a1/2*, *miR-133b* and *miR-206* with MFE of −25.9, −29.2 and −28.3 kcal), *lncRNA20194* (*miR-133b* and *miR-208* with MFE of −26.2 and −28.3 kcal, respectively), *lncRNA21817* (*miR-133b* and *miR-206* with MFE of −27.3 and −27.2 kcal, respectively), *lncRNA40141* (*miR-133b*, *miR-206* and *miR-208* with MFE of −25.8, −25.2 and −26.1 kcal), and *lncRNA60660* (*miR-206* with MFE of −25.1 kcal).

### 3.3. lncRNAs Expression in Gilthead Sea Bream Muscle

#### 3.3.1. lncRNAs Expression in Fast Muscle of Fingerlings, Juveniles, and Adults of Gilthead Sea Bream

In order to identify the possible roles of the selected lncRNAs in the development of the fast skeletal muscle of gilthead sea bream, their expression levels were studied under different in vivo and in vitro conditions. First, lncRNAs expression was analyzed on fast skeletal muscle from fish in different ontogenetic stages, expanding from fast-growing (fingerlings and juveniles) to slow-growing stages (mature/adult) (Figure 3). The majority of lncRNAs studied had a lower expression during the mature stage (*lncRNA20194*, *lncRNA21817, lncRNA02328, lncRNA54283, lncRNA60660*, *lncRNA31317*, and *lncRNA40141*) (Figure 3A–D,F–G,J) with a significant reduction between 77 and 34%. It was also found that *lncRNA62925*, *lncRNA43061*, *lncRNA14696,* and *lncRNA05337* showed no significant changes in expression between stages (Figure 3E,H–I,K). Only *lncRNA16861* had a significantly higher expression during juvenile and mature stages with an increase of 112 and 91%, respectively (Figure 3L).

#### 3.3.2. lncRNAs Tissue Screening

**Figure 4:**
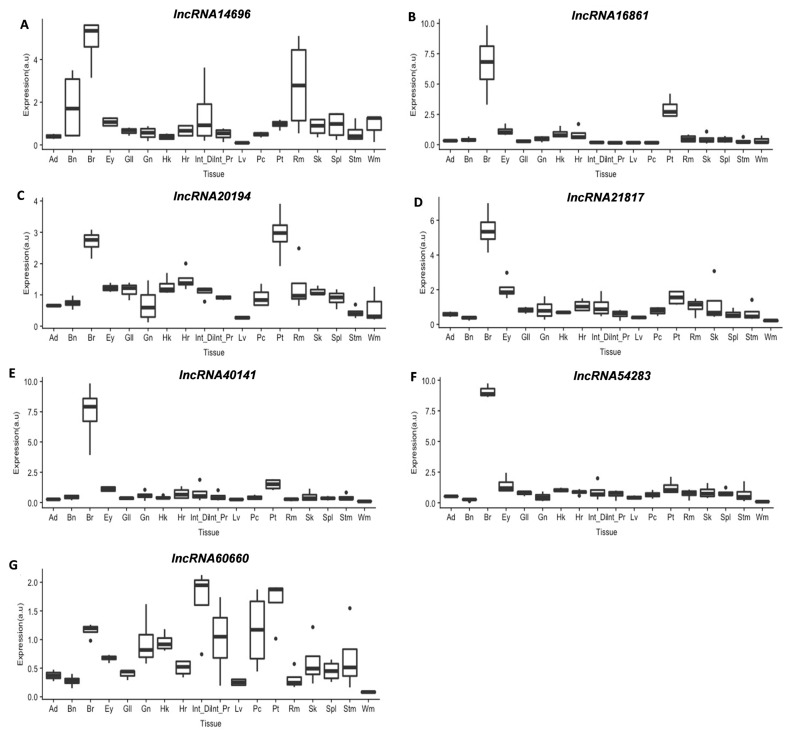
Boxplots showing lncRNAs expression in different gilthead sea bream tissues (*n* = 4) (**A**–**G**): Ad (Adipose tissue), Bn (Bone), Br (Brain), Ey (Eye), Gl (Gills), Hk (Head kidney), Hr (Heart), Int_Di (Distal Intestine), Int_Pr (Proximal Intestine), Lv (Liver), Pc (Pyloric caeca), Pt (Pituitary), Rm (Slow muscle), Sk (Skin), Spl (Spleen), Stm (Stomach), and Wm (Fast muscle). Gene expression is indicated as arbitrary units (a.u). Outliers are presented as points.

The analysis of lncRNAs expression in 18 different tissues showed that they were not exclusive of fast skeletal muscle (Wm) but detected in all tissues analyzed (Figure 4). Interestingly, except for *lncRNA14696*, the fast skeletal muscle was among the tissues with lower lncRNAs expression. In all cases, the slow muscle (Rm) showed a significantly higher expression than the fast skeletal muscle (Figure 4). It is also interesting that while *lncRNA14696*, *lncRNA20194*, and *lncRNA60660* (Figure 4A,C,G) had variable expression among tissues, *lncRNA16861*, *lncRNA21817*, *lncRNA40141*, and *lncRNA54283* had a much higher expression (5- to 7-fold change compared to fast muscle) in the brain (Figure 4B,D–F).

#### 3.3.3. lncRNAs Expression in Response to Nutrition

**Figure 5:**
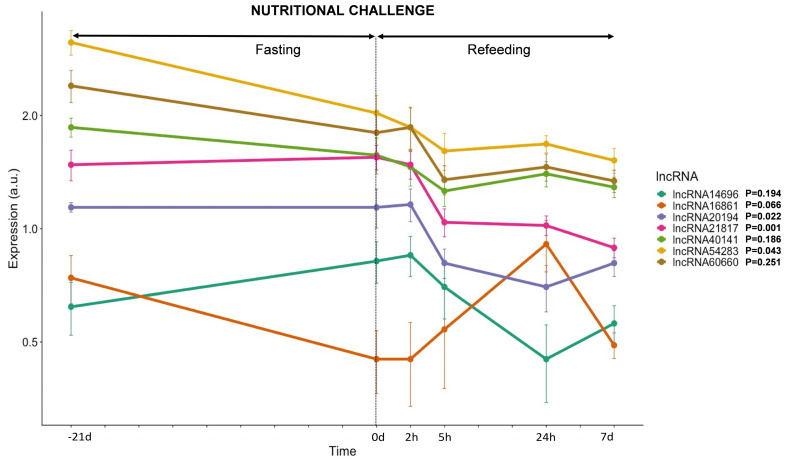
LncRNAs transcription in response to a nutritional challenge. Plot showing gene expression of 7 lncRNAs in response to 21 days of fasting followed by 7 days of refeeding. Gene expression is expressed as average ± SD (*n* = 6) of arbitrary units (a.u). The statistical effect of nutrition is indicated for each lncRNA analyzed.

The regulation of lncRNAs transcription by the gilthead sea bream fast skeletal muscle in response to changes in the nutritional status was also analyzed. Therefore, fish were fasted for 21 days and then refed to satiation for 7 days (Figure 5). The *lncRNA16861*, *lncRNA54283*, *lncRNA60660,* and *lncRNA40141* decreased their expression between 40 and 25% after 21 days of food deprivation, while *lncRNA20194* and *lncRNA21817* did not change, and *lncRNA14696* increased a 32% (Figure 5). Almost all lncRNAs, except for *lncRNA16861*, decreased their expression and kept a low transcription during the 7 days of refeeding, ranging between 50 and 30% reduction compared to pre-fasting values. Only *lncRNA16861* showed a significantly high transcription 24 h after refeeding started (23% increase compared to pre-fasting values), with a non-significant reduction of 35% by the end of the refeeding period.

#### 3.3.4. lncRNAs Expression during Myogenesis

**Figure 6:**
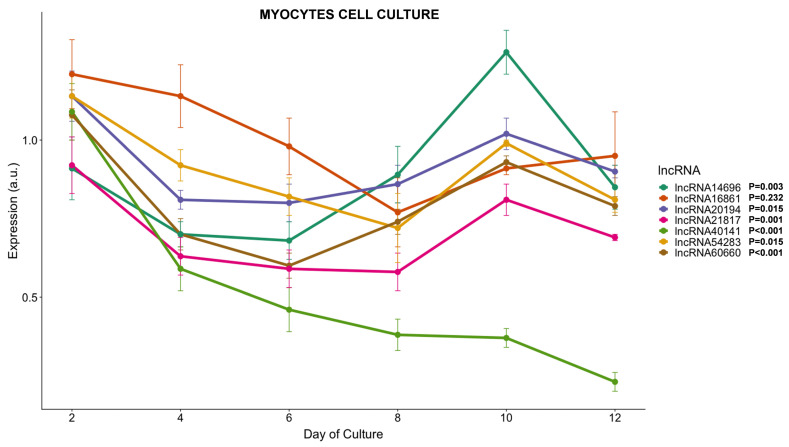
LncRNAs transcription during myocytes cell culture development. Plot showing gene expression of 7 lncRNAs during gilthead sea bream fast skeletal muscle myocytes cell culture. Gene expression is expressed as average ± SD (*n* = 6) of arbitrary units (a.u). The statistical effect of fasting and refeeding is indicated for each lncRNA analyzed.

In order to study the role of the lncRNAs in the myogenesis process in more detail, their expression was analyzed during the course of a primary myocyte culture, from the proliferative stage (days 0 to 4) and through the complete differentiation process (day 4 to 12). All lncRNAs analyzed except *lncRNA16861* (which showed a more stable expression during the whole culture) had a 20–35% decrease in their transcription between days 4 and 6 (Figure 6), concomitant with the myoblast proliferation phase. Until day 6, lncRNAs *lncRNA20194*, *lncRNA60660*, *lncRNA21817*, and *lncRNA54283* kept low transcription profiles (15–35% lower compared to the beginning of the culture). Between days 8 and 12, when myoblasts fuse to form myotubes and their maturation, several lncRNAs started to increase their transcription again (Figure 6). *LncRNA20194*, *lncRNA60660*, *lncRNA21817,* and *lncRNA54283* recovered 90% of the transcription compared to day 2; *lncRNA16861*, which recovered 85% of the day 2 transcription; and *lncRNA14696*, which even had an increase of 40% compared to day 2 values (Figure 6). The *lncRNA40141* did not follow any of those patterns and steadily continued reducing its transcription until day 12, when it was 80% lower than at the beginning of the cell culture (Figure 6).

#### 3.3.5. lncRNAs Expression during Muscle Regeneration

**Figure 7:**
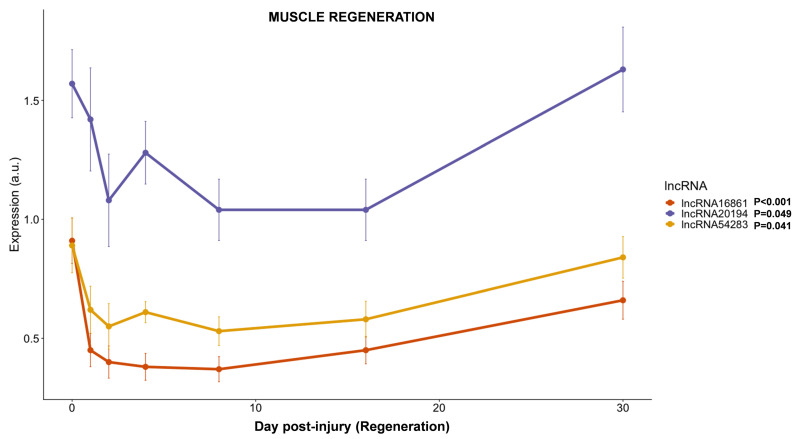
LncRNAs transcription in response to induced muscle injury. Plot showing the gene expression of three lncRNAs during 30 days of regeneration after an induced injury in the epaxial fast skeletal muscle of gilthead sea bream juveniles. Gene expression is expressed as average ± SD (*n* = 6) of arbitrary units (a.u). The statistical effect of muscle regeneration is indicated for each lncRNA analyzed.

The expression of three of the selected lncRNAs, the one predicted to interact with *myod1* (*lncRNA20194*), one of the lncRNA whose expression was higher in fingerlings (*lncRNA54283*), and the only lncRNA with higher expression in adults and juveniles (*lncRNA16861*), was studied during 30 days of regeneration after an induced injury in the epaxial fast skeletal muscle of juvenile gilthead sea breams. All three lncRNAs reduced their transcription by around 10 to 50% between days 1 and 2 after the injury (Figure 7). Except for a spike in the transcription of *lncRNA20194* at day 4 of regeneration, all lncRNAs analyzed kept a low transcription level (between 40 and 60% lower than pre-injury levels) for at least 16 days while recovering nearly normal values after 30 days of regeneration (Figure 7).

#### 3.3.6. Correlation between lncRNAs and Genes Related to Muscle Development

In order to further understand the possible role of the selected lncRNAs on muscle development, their correlation with muscle-related genes in the same samples of the regeneration experiment and the myocytes cell culture was estimated [18,71] (Table 2).

Interestingly, the great majority of lncRNAs analyzed (except for *lncRNA16861*) were positively correlated to *dock5* (r = 0.40–0.70) (Table 2). Beyond *dock5*, several correlations between lncRNAs and muscle related genes were found: *lncRNA14696* correlated to *mef2c* (r = 0.4) and *crkl* (r = 0.4); *lncRNA16861* correlated to *cav3* (r = −0.4) and *mymk* (r = −0.4); *lncRNA20194* correlated to *myod1* (r = 0.6), *myog* (r = −0.4), *myf6* (r = 0.4), *mymx* (r = 0.6), *mymk* (r = 0.3), *crk-a* (r = 0.4), and *crk-b* (r = 0.4); *lncRNA21817* correlated to *myod2* (r = −0.4), *myog* (r = −0.5), *cdh15* (r = −0.4), *mymx* (r = −0.4), *crk-a* (r = 0.4), and *crk-b* (r = 0.4); *lncRNA40141* correlated to *myod1* (r = 0.5), *myod2* (r = −0.5), *crk-a* (r = 0.6), *crk-b* (r = 0.8), and *crkl* (r = 0.4); *lncRNA54283* correlated to *myod1* (r = 0.3), *cav3* (r = −0.6,) *mymx* (r = 0.3), *crk-a* (r = 0.5), *crk-b* (r = 0.6), and *crkl* (r = 0.4); and *lncRNA60660* correlated to *myog* (r = −0.4), *myf6* (r = −0.4), *crk-a* (r = 0.7), *crk-b* (r = 0.4), and *crkl* (r = 0.6). The gene expression of *myod1* in the regeneration experiment and the myocytes cell culture is presented in the Supplementary File S5.

## 4. Discussion

In the present work, we took advantage of the existing transcriptomic data of gilthead sea bream fast skeletal muscle to focus on the non-coding genes, which have traditionally been overshadowed by the protein-coding genes. Because of the low expression of many lncRNAs, we used the normalized data published by Garcia de la serrana et al. (2012) from juvenile and adult gilthead sea breams’ fast skeletal muscle [55] to detect low expressed lncRNAs. By using this approach, 290 lncRNAs were identified in the fast skeletal muscle and were differently distributed between juveniles and adults. A greater number of lncRNAs was found in the juvenile gilthead sea bream transcriptome than in adults (209 compared to 17), which is a suggestion that the vast majority of lncRNAs detected play an important role in regulating muscle growth during that stage.

Primer design for the selected lncRNAs proved to be a challenge, with great difficulties finding primers with no self-dimer and cross-dimmer interactions or forming hairpins with themselves. These problems in primer design are likely to be the result of the different evolutionary pressures that act over the lncRNAs. While mRNAs are linear in structure to be translated, most of lncRNAs functions are derived from their three-dimensional structure; therefore, lncRNAs tend to form double strands within their sequence [24,72]. This increases the probability of two primers forming dimers and hairpins, increasing the difficulty of primer design. In addition, some lncRNAs were so poorly expressed that it was not possible to amplify them even from pure cDNA. Due to both obstacles mentioned, 8 of the 20 pre-selected lncRNAs could not be amplified since it was not possible to design suitable primers for them, or their expression was so low that it was not correct to analyze it by qPCR.

It is interesting to notice that four of the lncRNAs studied had a much higher transcription in the brain than in any other tissue, and even in those cases where lncRNAs were also expressed in many other tissues, the brain appeared to be one with a higher level of transcription. This should not be a surprise since previous studies reported that lncRNAs seem to be very important for the development of the neural system [29], with some researchers pointing out that around 40% of lncRNAs can be found to be highly expressed in the brain [73]. The expression in skeletal muscle was always very low, which might be in contradiction to an important role in muscle development. However, this is not necessarily the case for lncRNAs that are, in general, very low expressed [15]. Besides, all lncRNAs showed higher expression levels in slow muscle than in fast muscle. The difference between these two types of muscles could be because slow muscle is a metabolically and functionally more active tissue than fast muscle [74], and the role of lncRNAs in this tissue is likely to be more relevant. Nevertheless, further investigation will be necessary to understand the differences between the two tissues in the context of lncRNAs functions better.

The majority of lncRNAs showed similar patterns of regulation in response to different physiological contexts. For instance, most of them showed few changes in response to fasting but decreased shortly after refeeding started. Similarly, the three lncRNAs analyzed in the fish injury model were also downregulated shortly after muscle regeneration started. The correspondence in the regulation between lncRNAs is likely the results of the process of selection that was followed, in which it was prioritized those lncRNAs with higher expression in fingerlings and juveniles. The rationale behind this decision was to find lncRNAs with relevant roles in muscle growth and development. Then, those most expressed in the stages in which the fish growth rates are higher (fingerling and juvenile stages) had to be selected. Therefore, it is not unusual that those lncRNAs with the highest expression in the fingerling stage appeared to have similar patterns of transcription, while the only lncRNA selected with the lowest expression in fingerling (*lncRNA16861*) tended to have a different expression profile during fasting-refeeding but not muscle regeneration. The role played by these lncRNAs can be hypothesized from their expression profile during in vitro culture of myocytes. All lncRNAs, except for *lncRNA16861*, significantly changed their expression during the cell culture. Most of the lncRNAs were downregulated in the proliferative phase while increased again during the differentiation. These results suggest that their downregulation is important to maintain the proliferation of the myoblast, while their increase might be necessary for the transition to differentiation. Only *lncRNA40141* appeared to continuously decrease during myogenesis, suggesting that its inhibition might be important for the progression of the myogenic program. The downregulation of lncRNAs to promote proliferation and their increase to trigger the differentiation seems to fit with the results obtained in the in vivo experiments since the majority of lncRNAs were also decreased during proliferative stages while increased during fusion events. For instance, several studies in teleost fish demonstrated that after a period of food deprivation, when food intake is restored, there is an increase in proliferative muscle markers followed much later by an increase in differentiation [48,75,76,77]. Likewise, after a muscle injury, there is early activation of the satellite cells that are attracted to the injured zone and stimulated to proliferate, while later, during the regeneration of the injury, they fuse to form new fibers [78,79]. Similar roles in regulating muscle development were described for lncRNAs in humans, such as *Neat1* and *Lnc-31* [31,80] promoting proliferation, and *Myoparr*, *Munc,* and *LncMyod* promoting differentiation [35,81,82]. Our correlation analysis suggests the possibility that lncRNAs action is performed regulating the transcription of, at least, some of the genes controlling myogenesis progression, such as *myog*, *myod1,* or *myf5* [83,84], whose expression correlated positively with some of the lncRNAs analyzed. It is also interesting that all lncRNAs, except for *lncRNA16861* (only lncRNA with high expression in adult fish), correlate positively with *dock5* and, more than half of them to crk, both genes known to be involved in myoblast fusion [11]. These results also fit with the hypothesis that the lncRNAs identified are necessary to increase their transcription during differentiation, where myoblast fusion is a crucial step.

**Figure 8:**
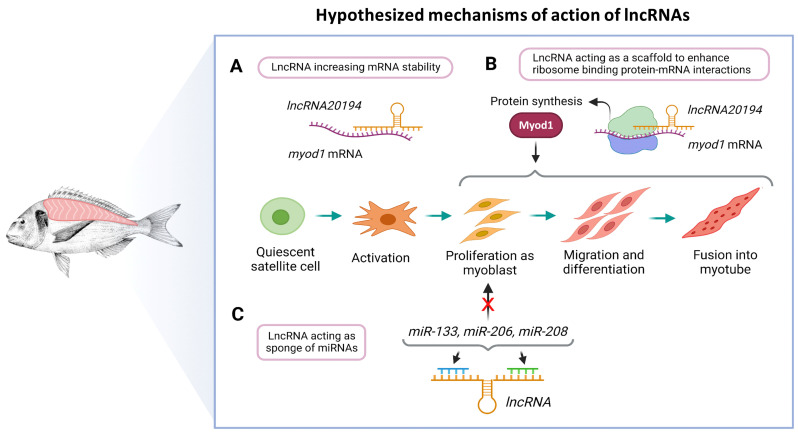
Hypothesized mechanisms of action of lncRNAs in the fast skeletal muscle of gilthead sea bream. The *lncRNA20194* might increase *myod1* mRNA stability (**A**) or act as a scaffold to enhance ribosome binding protein-mRNA interaction and thus promote Myod1 synthesis and function (**B**). The lncRNAs could also act as a sponge of miRNAs that are known to enhance myoblast proliferation, thereby regulating this step of myogenesis (**C**). These hypotheses are derived from the bioinformatic analyses.

The lncRNAs have multiple mechanisms to regulate gene transcription at very different levels [20]. Therefore, with the present data, it is not possible to establish the exact mechanisms of action. However, we studied two possible mechanisms of regulation, their interaction with mRNAs and miRNAs. The great majority of lncRNAs have weak interactions with mRNAs from genes known to be related to muscle development (ndG < −0.10). Only *lncRNA20194* showed a strong interaction with *myod1* (ndG = −0.17), as well as had a positive correlation with *myod1* (r = 0.6; *p* < 0.001). Although no formal mechanism of action can be proposed with the present data, one possible way of regulation could be a direct interaction of *lncRNA20194* with *myod1* increasing its function, for instance, stabilizing its mRNA or acting as a scaffold to enhance ribosome binding protein-mRNA interactions (Figure 8A,B) [85,86]. This hypothesis is also consistent with the cellular location of *lncRNA20194* since it was predicted to be in the cytoplasm (probability of 0.82) [87]. These results strongly suggest that the *lncRNA20194* is a promising candidate for further research.

The second mechanism of action could be the interaction with miRNA acting as sponges. Sponges lncRNAs can act as a decoy for one or multiple miRNAs, preventing them from binding to their targets [22]. All lncRNAs were able to bind some of the best-known miRNAs that regulate muscle development (*miR-1*, *miR-133a*, *miR-133b*, *miR-499*, *miR-206,* and *miR-208*). The miRNAs used to study their interaction with lncRNAs can either promote proliferation (*miR-206*, *miR-208,* and *miR-133*), differentiation (*miR-1*), or fiber contractile phenotype (*miR-499*) [88,89,90]. Some of the predicted interactions between lncRNAs and miRNAs were relatively strong (MFE < −25 kcal), but *miR-133*, *miR-206*, and *miR-208* were found in all cases. These results and the proposed hypothesis also fit with the results in the expression profiles observed in the in vivo experiments and throughout the myocytes cell culture since the lncRNAs downregulation happened during proliferation phases. If acting as sponges, their downregulation would lead to an increase in *miR-133*, *miR-206*, and *miR-208*, thus promoting proliferation (Figure 8C).

In order to confirm these hypothesized mechanisms of action of lncRNAs in the fast skeletal muscle of gilthead sea bream, further studies would be necessary. The suppression or overexpression of the lncRNAs would help to unravel their specific role in the regulation of myogenesis. However, transfection of primary myoblast cultures has yet to be developed in this species. Another drawback would be that there are no fish skeletal muscle cell lines, which for the study of lncRNAs would even have to be a specific gilthead sea bream cell line. In order to verify the interaction between *lncRNA20194* and Myod1 protein, it would be useful to perform, for example, a crosslinking immunoprecipitation combined with high-throughput sequencing (CLIP-seq) [91], which needs an antibody that works to immunoprecipitate the Myod1 protein. The investigation of the lncRNA mRNA and lncRNA miRNAs interactions will also pose a great challenge since the methods to decode RNA-RNA interactions, such as the ligation of interacting RNA and high-throughput sequencing (LIGR-seq), the psoralen analysis of RNA interactions and structures (PARIS), or the sequencing of psoralen-crosslinked, ligated, and selected hybrids (SPLASH), have never been tested in fish [92,93]. Finally, regarding the cellular localization of lncRNAs, RNA in situ hybridization [94] would be a valuable tool to confirm the results obtained from the bioinformatic analyses. Therefore, these subsequent functional studies on the lncRNAs will increase our understanding of the complex networks involved in regulating gilthead sea bream muscle growth and development.

## 5. Conclusions

The present work represents a first attempt to identify relevant lncRNAs for the development and growth of gilthead sea bream fast skeletal muscle. We found 290 lncRNAs expressed in fast skeletal muscle and identified seven that were differentially regulated according to the physiological context. The majority of lncRNAs studied were downregulated in those stages in which myoblast proliferation was more active and increased during fusion. The bioinformatic analysis suggested two possible mechanisms of action: first, by acting as sponges of *miR-133*, *miR-206*, and *miR-208*, important in promoting proliferation; secondly, by interacting with *myod1* mRNA, one of the myogenic regulatory factors regulating myogenesis. These results will serve as important resources for future studies that further investigate their ways of action and roles in muscle growth and development of gilthead sea bream.

## Figures and Tables

**Table 1:** Bioinformatic predictions of lncRNA interactions with muscle-related mRNAs and miRNAs and subcellular location.

LncRNAs	mRNA	ndG	miRNA	MEF (kcal)	Subcellular Location	Probability
*lncRNA14696*	*lamtor2 (ENSSAUG00010013916)*	−0.087	*miR-133a1/2*	−25.9	Nucleus	0.93
			*miR-133b*	−29.2		
			*miR-499*	−20.6		
			*miR-1*	−21.9		
			*miR-206*	−28.3		
			*miR-208*	−22.3		
*lncRNA16861*	*lamtor1 (ENSSAUG00010010852)*	−0.087	*miR-133b*	−23.2	Cytoplasm	0.89
			*miR-488*	−21.3		
			*miR-1*	−21.4		
			*miR-206*	−23.3		
			*miR-208*	−21.3		
*lncRNA20194*	*myod1 (ENSSAUG00010008630)*	−0.170	*miR-133a1/2*	−23.4	Cytoplasm	0.82
			*miR-133b*	−26.2		
			*miR-499*	−22.5		
			*miR-206*	−21.1		
			*miR-208*	−28.3		
*lncRNA21817*	NA	NA	*miR-133a1/2*	−23.3	Cytoplasm	0.70
			*miR-133b*	−27.3		
			*miR-499*	−23.4		
			*miR-206*	−27.2		
			*miR-208*	−21.6		
*lncRNA40141*	*eif4ebp1 (ENSSAUG00010019173)*	−0.085	*miR-133a1/2*	−22.4	Cytoplasm	0.46
			*miR-133b*	−25.8	Nucleus	0.26
			*miR-499*	−20.5		
			*miR-1*	−22.0		
			*miR-206*	−25.2		
			*miR-208*	−26.1		
*lncRNA54283*	*lamtor1 (ENSSAUG00010010852)*	−0.082	*miR-133b*	−23.4	Nucleus	0.49
	*pax7b (ENSSAUG00010013590)*	−0.080	*miR-206*	−24.3	Cytoplasm	0.37
			*miR-208*	−21.9		
*lncRNA60660*	NA	NA	*miR-206*	−25.1	Nucleus	0.67
					Cytoplasm	0.27

The strength of the predicted interactions with muscle-related mRNAs is indicated as length normalized free energy (ndG). The strength of the predicted interactions between lncRNAs against muscle-related miRNAs is indicated as minimal free energy (MFE). The probability of predicted subcellular cell location is also indicated. NA: not analyzed. *lamtor1* (late endosomal/lysosomal adaptor, MAPK and MTOR activator 1), *lamtor2* (late endosomal/lysosomal adaptor, MAPK and MTOR activator 2), *myod1* (myogenic determination factor 1), *eif4ebp1* (eukaryotic translation initiation factor 4E-binding protein 1), *pax7b* (paired box 7b).

**Table 2:** Correlation between gilthead sea bream lncRNAs and fast muscle-related genes.

lncRNAs	*myf5*	*myod1*	*myod2*	*myog*	*mef2c*	*myf6*	*cdh15*	*cav3*	*mymx*	*mymk*	*dock5*	*crk-a*	*crk-b*	*crkl*
*lncRNA14696*	0.2	0.1	−0.1	−0.2	0.4 *	−0.1	−0.1	0.3	−0.1	0.1	0.5 **	0.3	0.1	0.4 *
*lncRNA16861*	0	−0.1	−0.1	0.1	−0.1	0.1	0.1	−0.4 *	0.1	−0.4 ***	0.1	−0.02	0.3	−0.1
*lncRNA20194*	0.0	0.6 ***	0.1	−0.4 *	0.2	0.4 ***	−0.2	0.0	0.6 ***	0.3 *	0.4 **	0.4 *	0.4 *	0.2
*lncRNA21817*	0	0.1	−0.4 *	−0.5 **	0	0.1	−0.4 *	0.1	−0.4 *	0.1	0.6 **	0.4 *	0.4 *	0.3
*lncRNA40141*	−0.3	0.5 **	−0.5 **	−0.2	−0.2	−0.2	−0.1	−0.1	−0.2	−0.3	0.7 ***	0.6 ***	0.8 ***	0.4 *
*lncRNA54283*	−0.1	0.3 *	0	−0.1	0	0.1	0	−0.6 **	0.3 *	−0.1	0.5 ***	0.5**	0.6 ***	0.4 *
*lncRNA60660*	0.2	0.2	−0.2	−0.4 *	0.0	−0.4 *	−0.2	−0.1	−0.3	0.1	0.7 ***	0.7 ***	0.4 **	0.6 ***

Pearson correlation index between lncRNA and muscle-related genes expression. Significant correlations are indicated as follow: *p* < 0.05 *, *p* < 0.01 **, and *p* < 0.001 ***. *myf5* (myogenic factor 5), *myod1* (myogenic determination factor 1), *myod2* (myogenic determination factor 2), *myog* (myogenin), *mef2c* (myocyte enhancer factor 2c), *myf6* (myogenic factor 6), *cdh15* (cadherin 15), *cav3* (caveolin 3), *mymx* (myomixer), *myk* (myomaker), *dock5* (dedicator of cytokinesis 5), *crk* (Crk adaptor protein), *crkl* (Crk-like adaptor protein).

## Data Availability

The data presented in this study are available in the current article and its corresponding Supplementary material.

## Supplementary Materials

The following are available online at https://www.mdpi.com/2073-4409/11/3/428/s1. Supplementary File S1: Full cDNA sequences of 149 protein-coding genes and miRNAs known to be involved in muscle regulation; Supplementary File S2: Primers used in the Real-Time quantitative PCR analyses; Supplementary File S3: lncRNAs Ensembl IDs; Supplementary File S4: Figure showing the expression of a subgroup of lncRNAs in fast skeletal muscle from adults and juveniles sequenced by RNA-Seq; Supplementary File S5: Figures showing the expression of *myod1* along the regeneration experiment and the primary culture of myocytes.
