# Supplementary material for: The Emerging Role of Long Non-Coding RNAs in Development and Function of Gilthead Sea Bream (Sparus aurata) Fast Skeletal Muscle
Source: Cells. 2022 Jan 26;11(3):428. doi: 10.3390/cells11030428 (PMC8834446; doi:10.3390/cells11030428)
Supplement: Supplementary file 1 [file cells-11-00428-s001.zip › SUPPLEMENTARY FILES/Supplementary_File_2.pdf]

Table: Primers used in the Real-Time quantitative PCR analyzes.

lncRNAs	Primer sequences (5′-3′)	Ta (°C)	Efficiency (%)	Amplicon (bp)	Transcript ID (Ensembl) or Accession number (NCBI)
lncRNA14696	F: GATAGCGGCAGTTGGGTAAA<br>R: CAAAGAGAGGGAGAGCATCG	55	92.2	119	ENSSAUT00010014696
lncRNA16861	F: TGCAGTATGACTTGAGTGAAAAA<br>R: AGTTGCTGTTTTGCCTCCTT	54	102.4	79	ENSSAUT00010016861
lncRNA20194	F: GGACACGTAGACGCTGTGAA<br>R: CCTCCAAACAGTCGCTCAA	55	98.0	120	ENSSAUT00010020194
lncRNA21817	F: TGACAAAAACCTCAGGGATTG<br>R: CTTTGCGTGCTCTGTGGTAA	55	90.3	129	ENSSAUT00010021817
lncRNA40141	F: TTCCCTGTTTCTGGAGGATG<br>R: GGAGCATTTCCTGGTTGTGT	55	115.3	200	ENSSAUT00010040141
lncRNA54283	F: CTGCTGTGATCAGCGGTTT<br>R: AAAACGAGGTGGAAATGTGC	55	110.1	110	ENSSAUT00010054283
lncRNA60660	F: GCCTGACTCTCCATTGAAGC<br>R: ACAGCTCCCATCAGCTCACT	55	91.4	147	ENSSAUT00010060660
lncRNA02328	F: GAGGAAGGCTGGACTGTGAG<br>R: GTGGAGCTCTGCCCATAGAG	55	95.1	197	ENSSAUT00010002328
lncRNA62925	F: CGCATCCAATCAAAAGAGGT<br>R: CAGGATCTCACCCACAACACT	55	92.2	116	ENSSAUT00010062925
lncRNA31317	F: GTTGTTCAGGTTTCCCCTCA<br>R: GTCGGCGTTTTCTTTCCATA	55	112.5	153	ENSSAUT00010031317
lncRNA43061	F: TACGTTGGGAGGATGAGGAG<br>R: TGTACGCAGCCATAAAGCAG	55	200.2	199	ENSSAUT00010043061
lncRNA05337	F: ATGACGCCTCCATTCACTCCTCT<br>R: AGCTTTCCTGTGACAACGGTTGC	62	100.1	81	ENSSAUT00010005337
myf5	F: CCATCCAGTACATCGAGAGCC<br>R: ATCGCCCAAAGTGTCGTTCT	56	98.9	209	JN034420.1
myod1	F: TTTGAGGACCTGGACCC<br>R: CTTCTGCGTGGTGATGGA	60	96.4	139	AF478568
myod2	F: CACTACAGCGGGGATTCAGAC<br>R: CGTTTGCTTCTCCTGGACTC	60	101.2	149	AF478569
myog	F: CAGAGGCTGCCCAAGGTCGAG<br>R: CAGGTGCTGCCCGAACTGGGCTCG	68	96.7	182	EF462191.1
mef2c	F: ACAGCTTGTTGGTGCTGTTG<br>R: AGATAGCGCGAATCATGGAC	55	96.4	144	XM_030435338.1

myf6	F: CATCCCACAGCTTTAAAGGCA<br>R: GAGGACGCCGAAGATTCACT	60	100.6	151	JN034421
cdh15	F: GGCCTTCGTGGGTAGATAGG<br>R: TTTAACGTCGACCTGCTGTG	55	96.7	149	XM_030414763.1
cav3	F: CTGAGGGTGTGGACAAGGTCG<br>R: GTCAGGCCACGATAAACCCA	55	98.5	133	XM_030420899.1
mymx	F: TGCTGCGGTCCCTGGTTATC<br>R: ACTCCTGGGATCGAATGCGG	60	92.4	144	ENSSAUG00010011859
mymk	F: TTCACTGCGGTTTACCACGC<br>R: CCCACATAGAGAGAGCTGTGCTG	59	98.9	112	ENSSAUG00010019449
dock5	F: TCAACAGGCCCAGTAAATCC<br>R: GGGAAGCAGTTCCATCATTC	60	95.2	115	XM_030416547.1
crk-a	F: AACGCCCAGAACTCTGAAGG<br>R: ACCATGTCGCCCACCTCTAA	57	96.4	314	ENSSAUG00010004157
crk-b	F: ATGCCCGAGCCATTCAGAAG<br>R: TACCATGTCTCCCACCTCCAGA	59	95.8	77	ENSSAUG00010012872
crkl	F: TCCGCCTGGTATTTTGGACC<br>R: TGTGCTGCTTGGGTACCTGG	59	90.9	294	ENSSAUG00010019265
ef1a	F: CTTCAACGCTCAGGTCATCAT<br>R: GCACAGCGAAACGACCAAGGGGA	60	96.6	263	AF184170.1
rps18	F: GGGTGTTGGCAGACGTTAC<br>R: CTTCTGCCTGTTGAGGAACCA	60	97.7	163	AM490061.1
rpl27a	F: AAGAGGAACACAACTCACTGCCCCAC<br>R: GCTTGCCTTTGCCCAGAACTTTGTAG	60	97.7	160	AY188520.1

Ta: annealing temperature; F: forward; R: reverse; Amplicon: product size (base pairs).
