# Supplementary material for: The Emerging Role of Long Non-Coding RNAs in Development and Function of Gilthead Sea Bream (Sparus aurata) Fast Skeletal Muscle
Source: Cells. 2022 Jan 26;11(3):428. doi: 10.3390/cells11030428 (PMC8834446; doi:10.3390/cells11030428)
Supplement: Supplementary file 1 [file cells-11-00428-s001.zip › SUPPLEMENTARY FILES/Supplementary_File_4.pdf]

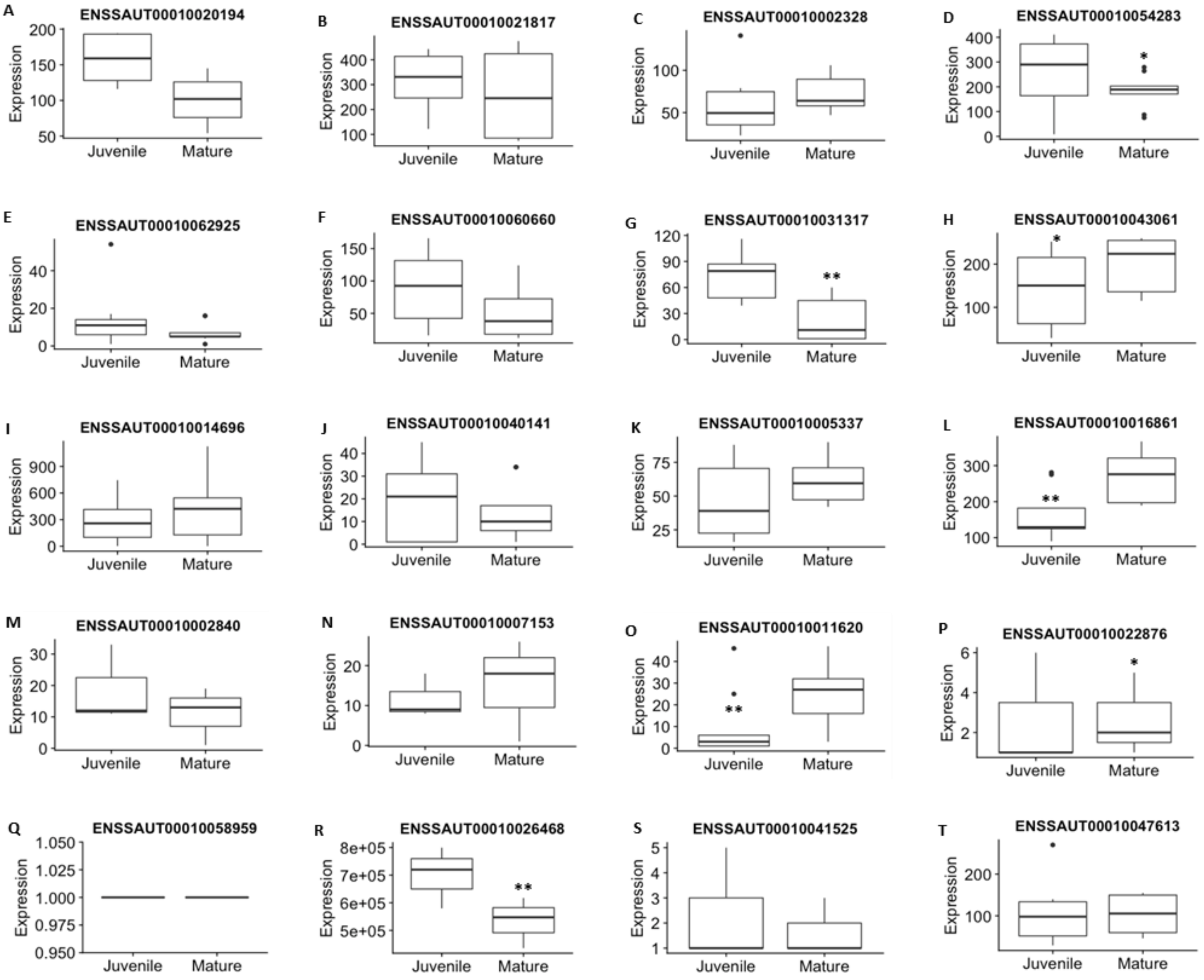

Figure. Expression of a subgroup of lncRNAs in fast skeletal muscle from adults and juveniles sequenced by RNAseq. Boxplot showing differences in RNAseq reads mapped against the gilthead sea bream genome (as DESEQ2 counts) between juvenile (n=3) and mature (n=3) gilthead sea bream's fast skeletal muscle. Statistical differences between groups are indicated as follow: *p<0.05, **p<0.01 and ***p<0.001.
