# Supplementary material for: The Emerging Role of Long Non-Coding RNAs in Development and Function of Gilthead Sea Bream (Sparus aurata) Fast Skeletal Muscle
Source: Cells. 2022 Jan 26;11(3):428. doi: 10.3390/cells11030428 (PMC8834446; doi:10.3390/cells11030428)
Supplement: Supplementary file 1 [file cells-11-00428-s001.zip › SUPPLEMENTARY FILES/Supplementary_File_5.pdf]

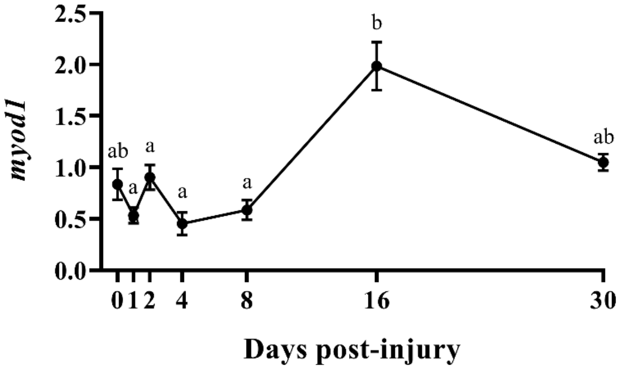

Figure. Relative gene expression of *myod1* along the regeneration experiment in white skeletal muscle from day 0 (time of injury) to day 30. Data are presented as means ± SEM (n=10). Different letters indicate significant differences ($p<0.05$).

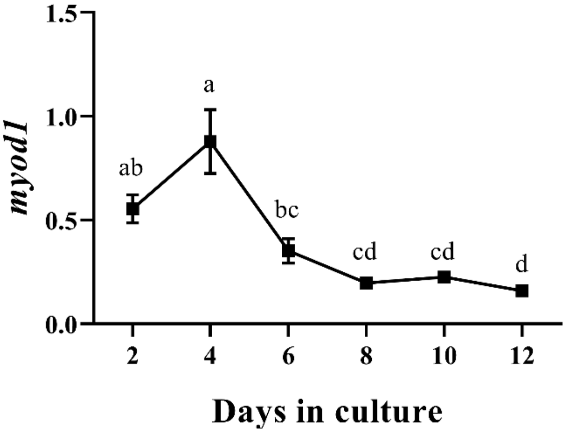

Figure. Relative gene expression of *myod1* along the primary culture of myocytes. Data are presented as means ± SEM (n=6). Different letters indicate significant differences ($p<0.05$).
